# Fluorinated Protein–Ligand Complexes: A Computational Perspective

**DOI:** 10.1021/acs.jpcb.4c01493

**Published:** 2024-06-17

**Authors:** Leon Wehrhan, Bettina G. Keller

**Affiliations:** Department of Chemistry, Biology and Pharmacy, Freie Universität Berlin, Arnimallee 22, 14195 Berlin, Germany

## Abstract

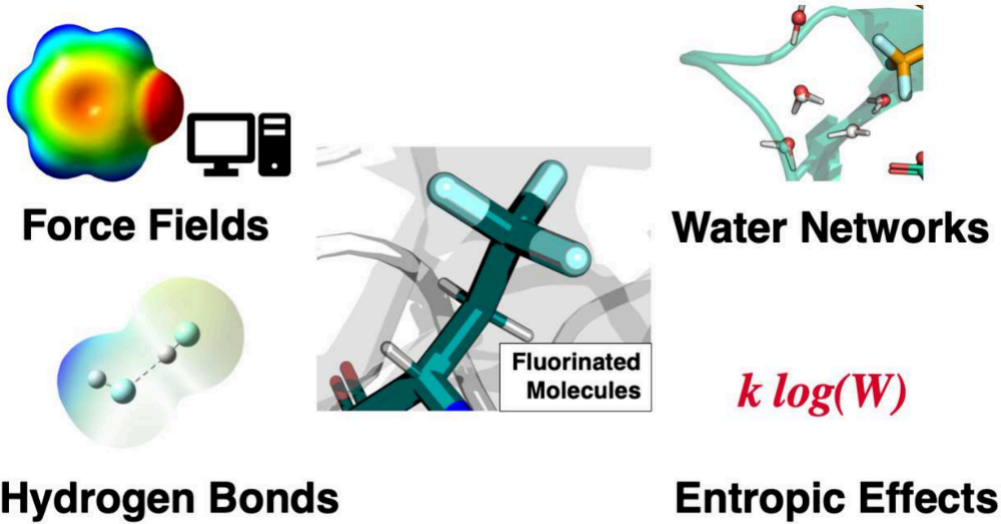

Fluorine is an element renowned for its unique properties. Its powerful capability to modulate molecular properties makes it an attractive substituent for protein binding ligands; however, the rational design of fluorination can be challenging with effects on interactions and binding energies being difficult to predict. In this Perspective, we highlight how computational methods help us to understand the role of fluorine in protein–ligand binding with a focus on molecular simulation. We underline the importance of an accurate force field, present fluoride channels as a showcase for biomolecular interactions with fluorine, and discuss fluorine specific interactions like the ability to form hydrogen bonds and interactions with aryl groups. We put special emphasis on the disruption of water networks and entropic effects.

## Introduction

Fluorine has an exceptional standing among the main group elements due to its remarkable reactivity in its elemental form and its extraordinarily high electronegativity. Even though inorganic fluorine is abundant on earth, fluorine is virtually absent from the organic compounds in living systems.^1,2^ Yet, fluorinated molecules are essential to medicinal chemistry, with a share of roughly 20% of fluoro-organic compounds in all globally registered pharmaceuticals.^3,4^ For example, fluorinated molecules have played a major role in the pursuit of anti-COVID19 drugs.^5^ Paxlovid, the first orally administered drug for the treatment for the coronavirus disease, is a combination of a fluorinated inhibitor of the coronavirus main protease and a nonfluorinated assisting compound.^5^

**Figure 1:**
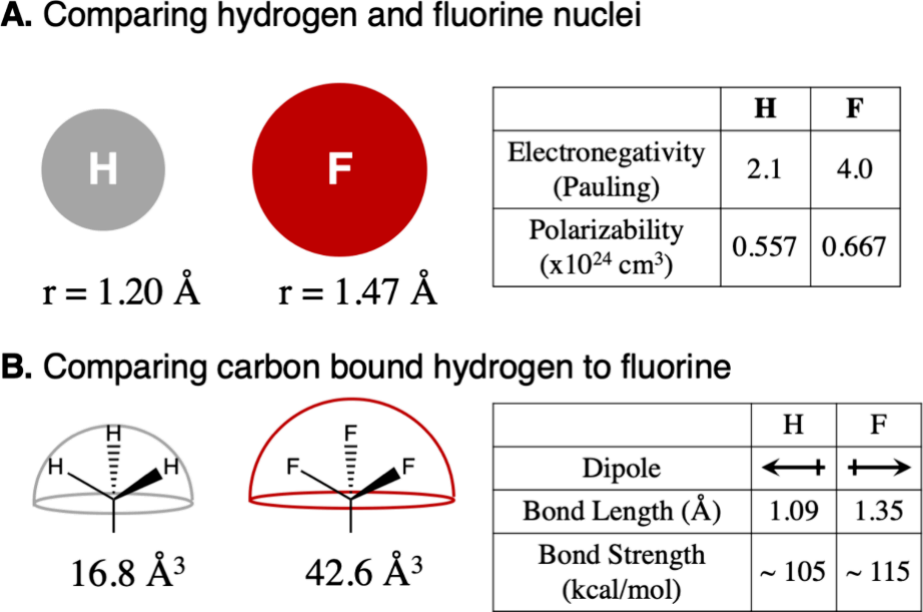
Comparison of basic properties of fluorine and hydrogen atoms (A) and of carbon bound fluorine vs carbon bound hydrogen (B). Adapted in part with permission from ref (7). Copyright 2020 Wiley.

The importance of fluorine for medicinal chemistry can be attributed to its exceptional potential to modulate the physicochemical properties of organic compounds.^1,6−8^ Fluorine is a small atom with a high electronegativity and low polarizability. With its van-der-Waals radius of 1.47 Å, fluorine occupies a smaller volume than typical organic substituents, like methyl, amino, or hydroxy groups, but is larger than a hydrogen atom (Figure 1). The C–F bond is strong (460–540 kJ/mol)^7^ and slightly longer than the C–H bond. Compared with the C–H bond, the C–F bond shows a reversed dipole moment, induced by the high electronegativity of fluorine. These properties make fluorine a powerful modulator of the lipophilicity, the p*K*_a_, or electrostatic potential of an organic molecule. Additionally, fluorine substituents can change the conformational preferences of organic molecules.^1,8^ Another pharmaceutically relevant effect is that fluorine can increase the metabolic stability of drug molecules.^9^ Finally, ^19^F, the only stable isotope of fluorine, has a nuclear spin of  and can thus be detected in NMR spectroscopy. It is a sensitive alternative to more commonly used nuclei like ^1^H, ^13^C, or ^15^N in NMR spectroscopy.^10^

Because fluorine substituents influence a wide range of molecular properties, the rational design of fluorinated molecules is challenging. Reference (11) gives an overview of noncovalent interactions of fluorine compounds. Several reviews cover the impact of fluorinated molecules in drug design and modern pharmaceuticals,^3−5,8^ as well as synthetic approaches and challenges of obtaining organic fluorinated molecules.^12−14^^19^F NMR spectroscopy in the context of fragment screening in drug discovery is reviewed in ref (15). Fluorinated amino acids and peptides are reviewed in refs (16−18).

Computational methods play an essential role in disentangling and understanding the relative magnitude of the often competing effects that a fluorine substituent can have on the molecular properties of the compound. Our goal here is to summarize how recent computational studies contributed to our understanding of fluorine’s role in protein ligand binding. We particularly focus on molecular simulations and include an overview of recent atomistic force fields for fluorinated substances. While the focus of this Perspective lies on molecular simulations using classical atomistic force fields, fluorinated protein ligands have also been studied with quantum chemical calculations, like those in ref (19) and ref (20).

In a protein environment, fluorine can act as a hydrogen bond acceptor but can also form contacts with nearby aryl groups.^21,22^ It has even been proposed that fluorine substituents do not need to interact with the protein directly but instead bind to the protein via water mediated contacts.^23,24^ Closely related to these water networks is the desolvation of fluorinated ligands during the binding process, which induces surprising enthalpy–entropy compensation effects.^25,26^ We hope to provide useful insight into how the relative importance of these effects can be measured by using molecular simulations.

## Results and Discussion

### Force Fields

**Figure 2:**
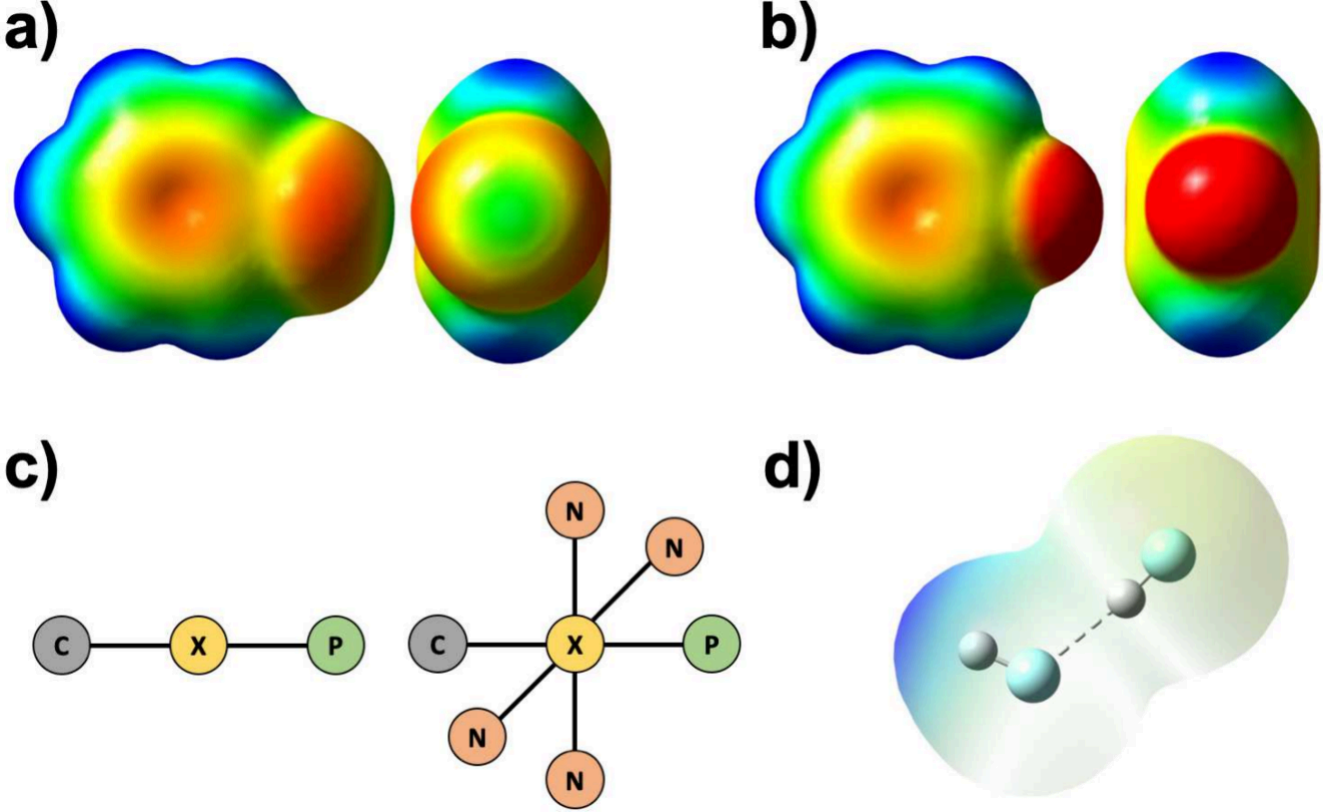
(a) Electrostatic potential of chlorobenzene, top view of the aromatic system, and view along the Cl–C bond. (b) Electrostatic potential of fluorobenzene, top view of the aromatic system and view along the F–C bond. (c) Molecular mechanics based models for covalently bound halogens: (left) positive extra point model; (right) 5-pseudoatom model with positive (P) and negative (N) extra point charges. (d) HF dimer in typical angled geometry. Electrostatic potentials were calculated from QM structure optimization at the MP2/aug-cc-pVTZ level of theory in Gaussian16. The electrostatic potentials are shown on the MP2 electron density surface with an isovalue of 0.0004 au. (a) and (b) show the electrostatic potential on a scale from −55 to +55 kJ/mol. (c) shows the electrostatic potential on a scale from −50 to +250 kJ/mol.

The accuracy of a molecular simulation critically depends on the underlying potential energy function, whose negative gradient is the molecular force field. The challenge in describing halogen substituents within the functional form of a molecular mechanics force field lies in balancing the Coulomb and the van-der-Waals interactions. Additionally, covalently bound halogen atoms may exhibit a highly anisotropic charge distribution, which consists of a negative charge belt in the direction perpendicular to the covalent bond and a region of diminished electron density on the opposite side of the covalent bond, called the σ-hole^27^ (Figure 2a). Due to this anisotropic charge distribution, an intermolecular interaction called a halogen bond may form, in which a lone pair on the neighboring atom binds to the σ-hole. The σ-hole decreases from iodine to fluorine (Figure 2.b), with bromine and chlorine featuring medium-sized σ-holes.

Most atom types in molecular mechanics force fields are modeled by using a single point charge, which creates an isotropic electrostatic potential around this atom. These atom types lack the ability to model the σ-hole and halogen bonds.^28,29^ The anisotropic charge distribution of covalently bound halogens can, however, be modeled by introducing a positive extra point (PEP) close to the halogen atom,^30^ or by a 5 pseudoatom scheme,^31^ where an extra four pseudoatoms are introduced to model the negative charge belt around the halogen (Figure 2c). Another approach is to tune the Coulomb and Lennard-Jones interactions of the halogen using cosine functions^29,32^

The anisotropic charge distribution around fluorine is particularly relevant in hydrogen fluoride, where it leads to an angled hydrogen bond geometry. Parameters for hydrogen fluoride which make use of an positive extra point charge and correctly reproduce the hydrogen bond geometry have been proposed in recent years^33,34^ (Figure 2d).

In fluorine bound to carbon, the electrostatic potential is almost isotropic around the fluorine substituent^35^ (Figure 2b), and therefore, the fluorine atom in carbon–fluorine substituents may be modeled with a single point charge. In fact, generic fluorine atom types with a single point charge and corresponding van-der-Waals parameters are included in traditional all-atom force fields like GAFF/GAFF2. However, the balance between Coulomb interactions and van-der-Waals interactions can vary significantly across different organic compounds. Parameters determined for model compounds may not seamlessly align with those of a protein force field. As a result, various customized parameter sets have been published for specific fluorinated compounds and fluorinated amino acids.

Multiple fluorinated amino acids have been added to the CHARMM36 protein force field and the CHARMM general force fields.^36^ Furthermore, a set of fluorinated aliphatic amino acids^35,37^ and a set of fluorinated tyrosine derivatives^38^ was added to the AMBER14SB protein force field. Using the implicit polarization charge scheme, parameters for fluorinated aromatic amino acids were added to the AMBER ff15ipq protein force field.^39^

Robalo et al.^37^ have focused on balancing the thermodynamic properties of fluorinated amino acids. Their parameter sets are based on point charges and the AMBER functional form,^35,37^ where the fluorine point charges are calculated based on the AMBER typical RESP fitting procedure. To account for the variations in the partial charge due to conformational changes in the amino acid, they determine a conformationally averaged partial charge by iteratively fitting point charge and sampling the conformational equilibrium. The parameters of the Lennard-Jones potential, which is used to model the van-der-Waals interactions in AMBER, are then optimized against the hydration free energy of CF_4_ and the molar volume of an equimolar CF_4_:CH_4_ mixture, to capture the hydrophobic effect of fluorine and packing constraints in the hydrophobic core of proteins. To model hydrogen bonds to partially fluorinated carbons, the authors additionally optimize the Lennard-Jones parameters of the hydrogen atom on these partially fluorinated carbons against the hydration free energy and molar volume of HCF_3_.

While ref (37) is concerned with fluorinated amino acids carrying one or two fluorinated carbons and focused on changing the nonbonded force field parameters of fluorine, it may sometimes be necessary to reparametrize the bonded force field terms as well. Träg and Zahn benchmarked dihedral parameters of the GAFF2 all-atom force field and found weaknesses when describing molecules with more than three adjacent fluorinated carbons.^40^

The examples above show that while traditional point charge based force fields are sufficient to model fluorine substituents, generic fluorine parameters have a limited scope and often need to be fine-tuned to the system at hand. These reparametrizations can be tedious. Automatic or semiautomatic processes could help to streamline the parametrization of fluorinated molecules in the future. One approach is applied in the open force field.^41−43^ The open force field iterations Parsley^42^ and Sage^41^ evade the use of atom types, required for traditional force fields, and use a process called native chemical perception instead that relies on querying functional groups with SMIRKS strings.^43^ This way, the force field is easily extendable without excessively inflating the complexity by adding large numbers of new atom types. Another recent promising approach is the Espaloma^44,45^ force field. The authors use graph neural networks to perceive chemical environments (by learning embeddings for atoms, bonds, angles, and dihedrals) and determine force field parameters based on QM calculations in a way that is completely end-to-end differentiable with respect to model parameters. In this way, developing customized parameters for fluorinated ligands can be automated by using standard neural network libraries. We note that accurate force field parameters are essential not only for molecular simulations but also for any computational method that requires an accurate molecular-mechanics based representation of the fluorinated molecules, such as molecular docking.

Having stressed the importance of selecting an appropriate force field in general and for fluorinated compounds in particular, our focus shifts to exploring specific interactions in the following sections. Before we explore the specific interactions and effects of fluorine in protein–ligand systems, we present fluoride channels as a case study of how proteins interact with fluorine, here in the form of a fluoride anion.

### Fluoride Channels (Flucs)

Fluoride channels (Flucs) are an interesting case study that showcases how proteins interact with fluoride anions. Flucs are membrane channels found in microorganisms, where they export toxic fluoride anions across the cell membrane.^46^ Their structure is unique, as the Flucs are expressed as homodimers, where the monomers are aligned in an antiparallel manner. Despite the antiparallel alignment, the monomers transport fluoride ions independently in the same direction, so that inactivating one of the monomers does not stop fluoride transportation through the other one. Flucs are exceptionally selective for fluoride over chloride and small cations. How this selectivity is achieved and how fluoride ions traverse the channel is subject to current research.^47−49^

Yue et al.^47^ studied the fluoride channel of *E. coli* (Fluc-Ec2) using the polarizable Drude force field and CHARMM36 additive force field for comparison. They employed replica exchange umbrella sampling to get a potential of the mean force of the fluoride position along the channel. They find energetic minima that align with fluoride positions observed in crystal structures and calculate permeation rates that match the experimental values well. The fluoride permeation relies on a network of different nonbonded interactions in the channel that compensate for the desolvation of fluoride. These interactions include hydrogen bonds between amino acid side chains, the protein backbone, or water and fluoride, ionic contacts to positively charged side chains, and anion−π contacts. The nonpolarizable force field overestimates the interactions and therefore leads to wrong permeation rates, which indicates that the polarizability plays an important role in the ion permeation process.

**Figure 3:**
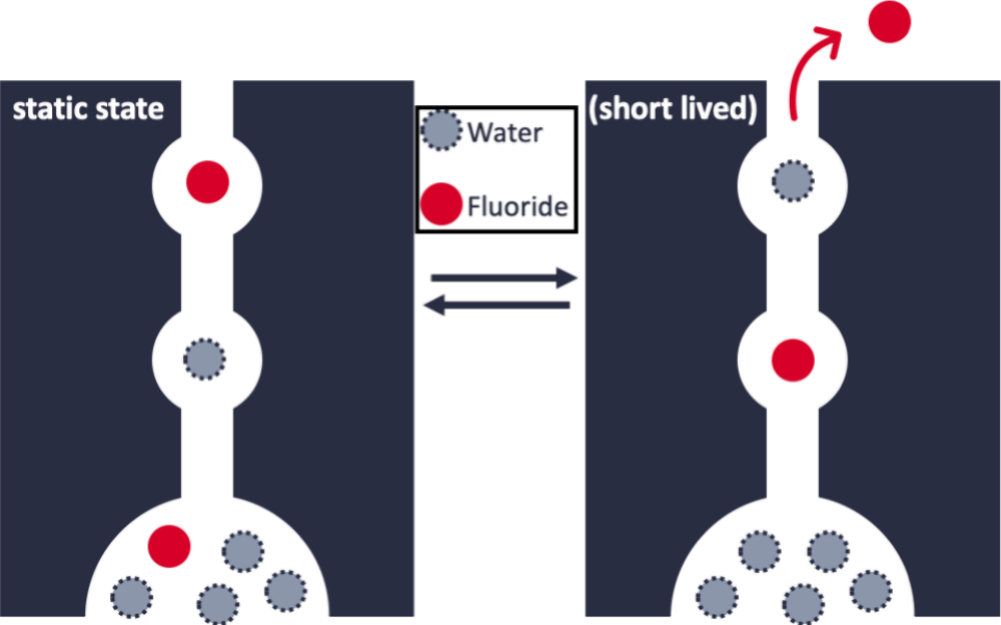
Fluoride ion transport in Flucs via the water mediated “knock-on” mechanism proposed by Zhang et al.^48^ One of internal sites are occupied by a fluoride ion in the static state. A second entering fluoride will expel the first fluoride via a short-lived state that eventually returns back to the static state. Adapted in part from ref (48). Available under a CC BY-NC license. Copyright 2023 Zhang et al.

Zhang et al.^48^ expand on this study by using solid state NMR and molecular dynamics simulations of the Flucs and mutated versions of the Flucs to investigate the permeation mechanism. They identify additional fluoride residence sites at the aqueous regions by the entrance and exit of the channel and discover, through molecular dynamics, that these sites can be explored by fluoride as well as by chloride. The authors also identify a structural loop that is important for channel gating. They propose a water mediated “knock on” mechanism for the fluoride permeation (see Figure 3). In this model, one of the two internal fluoride sites within the Fluc is occupied by a fluoride, while the other is occupied by water in the static state. An entering fluoride pushes the water into the next site, where the residing fluoride will be expelled from the channel. The state that is now formed is short-lived and will return to a static state.

This proposed mechanism has intriguing consequences for fluorine–protein interactions. As the fluoride ions inside the channel are likely to be unhydrated, the desolvation energy of 464 kJ/mol^47^ has to be compensated by interactions of the protein with the fluorine anion. These interactions are ionic contacts and hydrogen bonds, which are likely to be particularly stable, because of the negative charge of the fluoride ion. Moreover, anion−π interactions are observed, which are not as common as cation−π interactions and rely on the interaction of the anion with the positive edge of an aromatic system.

While fluoride anions are naturally different from fluorine in small molecule protein ligands, the case study explored here demonstrates how interactions of proteins with fluorine can counteract sizable magnitudes of energy, such as the desolvation of fluoride. Moreover, this case study demonstrates how molecular simulation methods can be utilized to study the interactions between fluorine and proteins. We now shift our focus to fluorine in small molecule ligands and its specific interactions.

### Effects Involving Aryl Groups

Fluorine has a strong influence on conjugated or aromatic π-electron systems in its vicinity due to its high electronegativity. When the fluorine is attached to an aryl group, as in most fluorinated pharmaceuticals,^3^ it acts as an electron-withdrawing group and reduces the electron density in the aromatic system. This weakens any π-stacking interactions the unfluorinated aryl might be involved in.^50,51^ When fluorinating tyrosine, the electron-withdrawing effect of the fluorine substituent leads to a decreased p*K*_a_ of the tyrosine hydroxyl group.^52^ Fluorinating anilines can have interesting behavior on their hydrogen bonding abilities, as monofluorination leads to weaker and less frequent NH···N hydrogen bonds while fluorination of 4X-anilines can increase the strength of these bonds.^53,54^

**Figure 4:**
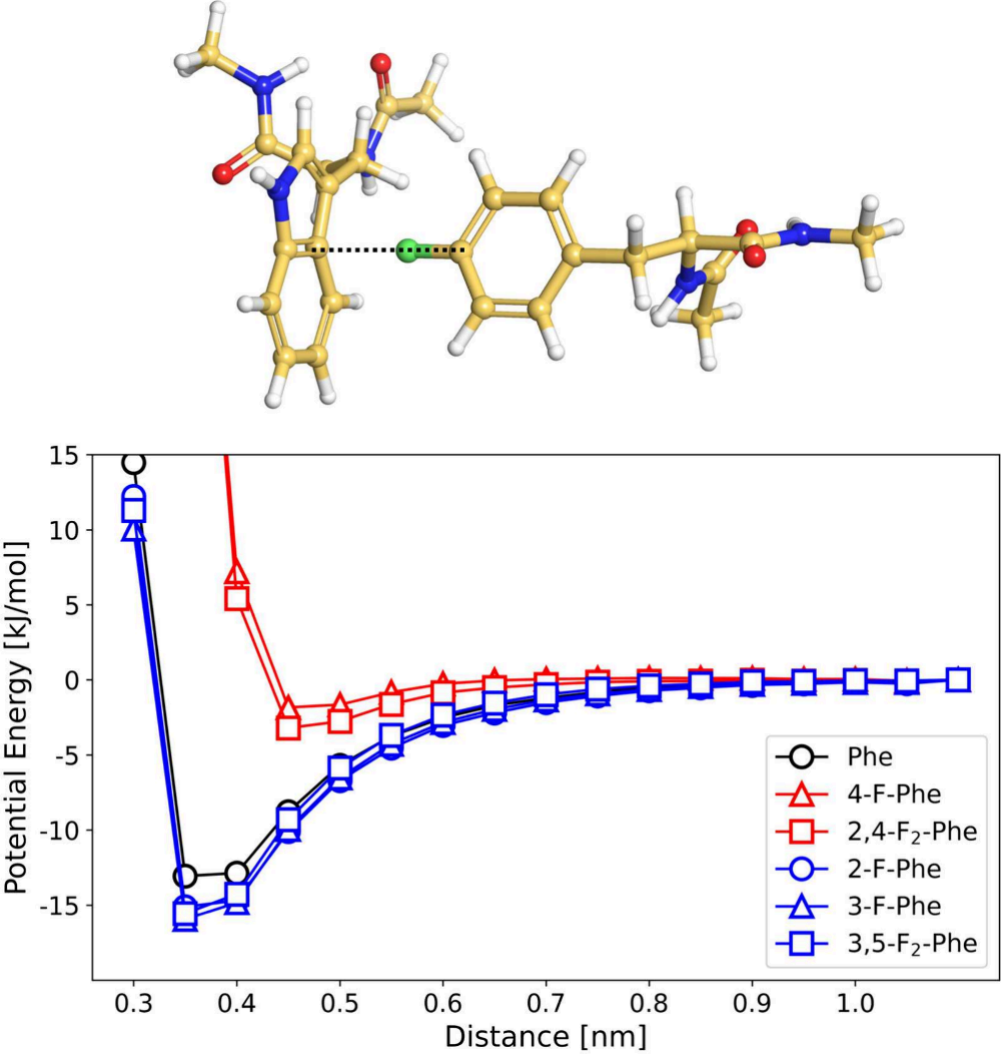
Potential energy of the T-shaped π interaction between variants of phenylalanine and the aromatic ring of tryptophan. The interaction energy was calculated using GAFF2 parameters. Adapted from ref (22). Available under a CC BY-NC license. Copyright 2023 Müll et al.

Fluorine can also directly interrupt a T-shaped π-interaction if it is introduced as a substituent on the aryl group that represents the T-stem (Figure 4). Müll et al.^22^ discovered that the preference of nonribosomal peptide synthetase for the natural substrate phenylalanine is 31 times higher than that for a singly fluorinated phenylalanine. But this preference is observed only when the fluorine substituent is positioned in the *para*-position, i.e., directly pointing toward the π-system of the aryl group that represents the T-bar. If phenylalanine is fluorinated in the *meta*- or *ortho*-position, the fluorinated phenylalanines are accepted as substrate with about the same likelihood as phenylalanine.

This drastic change in substrate activity can be explained through computational modeling, which reveals that the potential energy minimum of the T-shaped π-stacking interaction is almost completely erased if the partially positively charged hydrogen in the *para*-position of phenylalanine is substituted by a partially negatively charged fluorine atom. By contrast, fluorination at other positions of the phenyl ring besides the *para*-position slightly lowers the energy minimum and thereby stabilizes T-shaped π-stacking interaction. This is likely caused by the electron-withdrawing effect of the fluorine substituent in the *meta*- or *ortho*-position, which increases the partial charge on the hydrogen atom in the *para*-position. It is worth pointing out that the loss of the π-stacking interaction is a sizable enthalpic effect, as it decreases the interaction strength (in the model system in Figure 4) by about 10 kJ/mol.

Fluorine’s impact on aromatic systems is particularly important in the context of drug discovery, given its frequent occurrence attached to aromatic systems in pharmaceuticals. Here, we discussed the indirect effect fluorine may have on the interactions of aromatics in protein systems, and we demonstrated how fluorine can directly disrupt aromatic interactions with proteins. While in this example fluorine unfavorably impacts the binding affinity, it is reasonable to assume that fluorine may also have a favorable effect on binding affinities through direct interactions. In the next section, we will cover a particularly important type of such an interaction, hydrogen bonds to fluorine substituents.

### Hydrogen Bonds to Fluorine Substituents: Donor’s Last Resort?

Hydrogen fluoride in the gas phase and in aqueous solution forms strong hydrogen bonds. Most notably, the hydrogen bond within the bifluoride anion, FHF-, is the strongest known hydrogen bond. With a dissociation energy of 161.5 kJ/mol,^55^ it is in fact so strong that it is disputed whether the bond should be counted as a hydrogen bond.^56^ By contrast, the hydrogen bonds to fluorine substituents in organic molecules are much weaker and their influence on the stability of protein–ligand complexes has been discussed controversially.^57−62^ Despite the high electronegativity of fluorine, fluorine substituents are weak hydrogen bond acceptors, which is attributed to fluorine’s low polarizability and low charge capacity.^61,62^ The hydrogen bond strength of C–F···H–O is between 6 and 10 kJ/mol, depending on the hybridization of the C atom.^58^ This is less than half of the typical strength of a hydrogen bond O···H–O, which is about 21 kJ/mol.^63^ Dalvit et al. therefore conclude that intermolecular hydrogen bonds with fluorine as acceptor only occur in environments shielded from water and void of other competing acceptors.^61^

**Figure 5:**
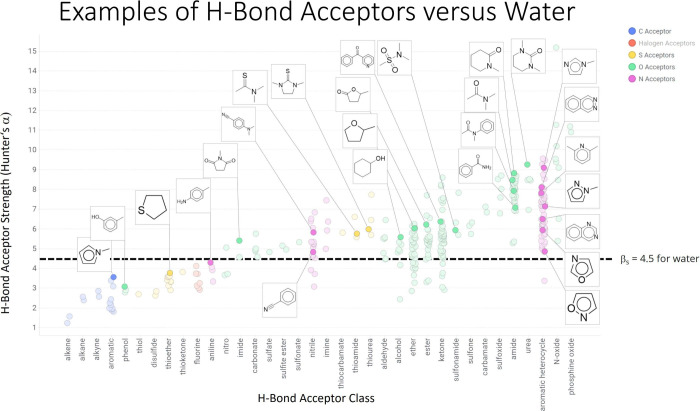
Hydrogen bond acceptor strength of common hydrogen bond acceptors in protein ligands found in medicinal chemistry. Reprinted with permission from Daryll McConnel.

Nevertheless, fluorine hydrogen bonds are frequently observed in protein–ligand complexes, particularly if the fluorinated ligand is a small organic molecule. Protein targets include FXIa,^64^ μ opioid receptor,^24^ YAP:TEAD protein–protein interaction,^65^ S1P receptor,^66^ Akt1,^67^ HIV protease,^68^ tyrosinase,^69^ Bruton’s tyrosine kinase,^70^ Janus kinases,^71^ and the SARS-CoV-2 main protease.^72−74^ Thus, hydrogen bonds with fluorine as an acceptor are by no means a marginal phenomenon in protein–ligand systems. Whether they are a driving force for the stability of a protein–ligand complex and can thus be exploited as a design element is, however, questionable. Compared to other typical hydrogen bond acceptors in protein ligands (Figure 5), fluorine ranks rather low. Interestingly, fluorine is an even weaker acceptor than water, meaning that replacing a water molecule as an acceptor from a protein donor with fluorine results in an enthalpic loss.

A recent survey^21^ of protein–ligand complexes in the protein data bank yielded more than 4000 complexes with fluorinated ligands. The authors identified hydrogen bond donors to the fluorine acceptor and evaluated the hydrogen bond interaction energy. When considering the difference in energy between the isolated hydrogen bonded structure and the donor and acceptor separated from each other, the hydrogen bond interaction energies range from 0 to −5.02 kJ/mol.

Fluorine was found to accept hydrogen bonds from OH, NH, and CH donors, with CH being the most frequent donor. As expected, the interaction energy depended on the donor–acceptor distance. However, it did not correlate with the F···H–X angle, which indicates that the hydrogen bond is not necessarily aligned with a lone pair of the fluorine acceptor. Moreover, the distances and angles of the fluorine hydrogen bonds did not coincide with the energy minima of the corresponding isolated hydrogen bonds. This leads to the conclusion that the enthalpic gain due to the fluorine hydrogen bonds is likely not the driving force of the binding affinity. The fluorine hydrogen bonds probably form in addition to stronger interactions that stabilize the ligand in the binding pocket. This causes the authors to taunt fluorine as “donor’s last resort”.

**Figure 6:**
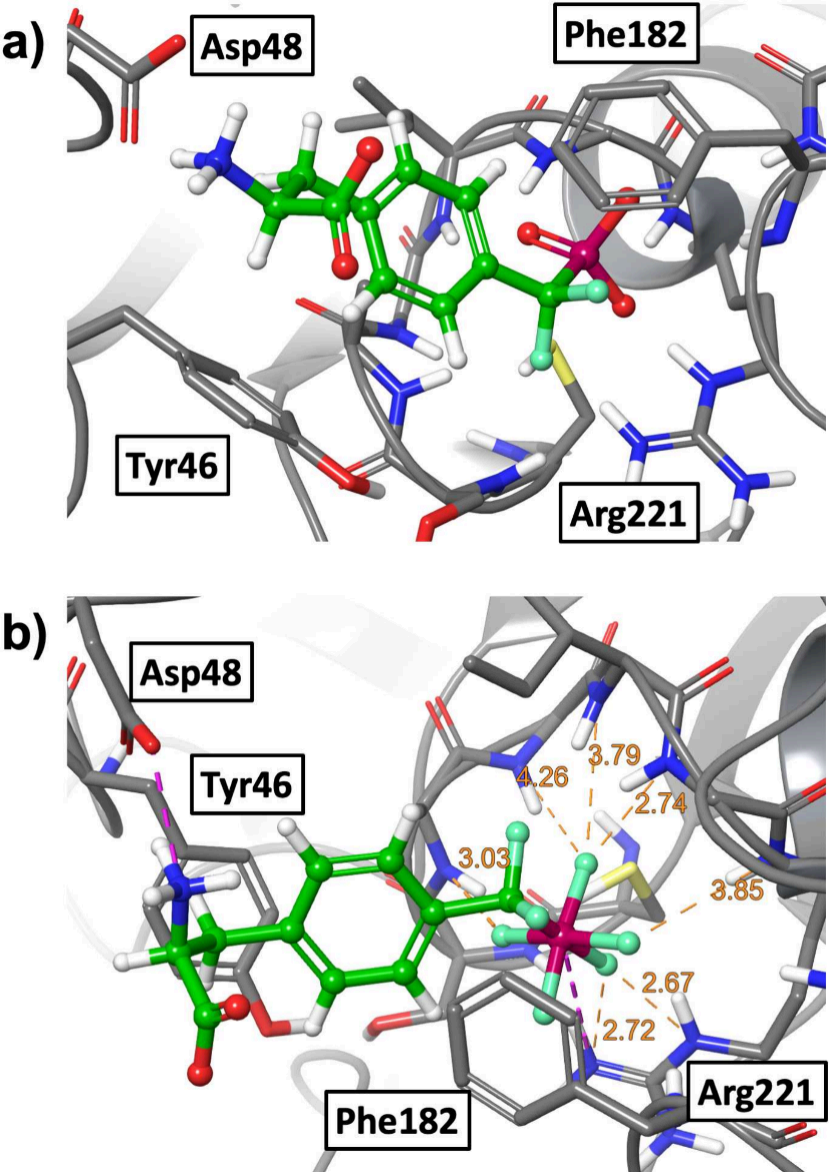
(a) Phosphotyrosine mimetic amino acid with phosphate headgroup in the main binding pocket of PTP1B. (b) Phosphotyrosine mimetic amino acid with the PF5 headgroup in the main binding pocket of PTP1B. Adapted from ref (75). Available under a CC BY-NC license. Copyright 2022 Accorsi et al.

The acceptor strength of fluorine atoms can be boosted considerably by incorporation into a larger functional group. For example, fluorine can replace oxygen in a phosphate group, generating fluorinated analogues of phosphate groups. Accorsi et al.^75^ used this strategy to improve the phosphotyrosine mimetic 4-phosphono(difluoromethyl)phenylalanine, which inhibits the tyrosine phosphatase PTP1B (Figure 6). The phosphate group in the original inhibitor is replaced by a pentafluorophosphato group (PF5), which improves the binding affinity from *K*_*I*_ = 1555 μM to *K*_*I*_ = 61 μM, where *K*_*I*_ is the inhibition constant measured in an enzyme inhibition assays. The pentafluorophosphato group has a valency of six, isoelectronic to a hexafluorophosphate anion, and a charge of −1. The expected charge state of a phopshate group is between −1 and −2 at pH 7. Thus, the increased binding affinity cannot simply be attributed to an overall increased Coulomb interaction between the positive binding pocket of PTP1B and the negatively charged functional group. Instead, computational docking and molecular dynamics simulations show that the PF5 headgroup sterically fits well into the binding pocket and forms several stable interactions between an arginine side chain and multiple protein backbone NH moieties. Similar fluorine specific interactions in the PTP1B binding pocket were also observed by Tiemann et al.,^76^ where a trifluoromethylsulfonamide headgroup is placed into the binding pocket.

In conclusion, heavy fluorination of the phosphor headgroup draws so much negative charge density to this essential part of the inhibitor that the interaction with the very positive binding pocket is likely to be significantly enhanced by direct fluorine hydrogen bonds.

### Water Networks

An interesting yet highly controversial question is whether fluorinated ligands can bind to proteins via water-mediated hydrogen bonds. In this scenario, the fluorine substituent is in contact with water molecules in the binding pocket rather than with the protein surface. The hypothesis then is that the partial negative charge of the fluorine substituent and fluorine’s capacity to accept hydrogen bonds structure the water network and thereby stabilize the ligand in the binding pocket. Computations of fluorinated amino acids and how they interact with water have shown that hydrogen-bond-like interactions between organic fluorine atoms and water molecules do occur and influence hydration free energies.^35,37,77^ Additionally, ultrafast fluorescence spectroscopy revealed that fluorinated amino acid side chains can slow down water motion on protein surfaces.^78^ These results suggest that fluorine substituents may indeed interact with proteins via water networks. However, detecting the change in the water network and disentangling the various interactions involved requiring atomistic simulations and detailed computational analyses, as we will showcase in the following examples.

Van der Westhuizen et al.^79^ analyzed the binding poses of a series of inhibitors of acetylcholinesterase using molecular docking and measured their activity with an enzyme inhibition assay. In one of the scaffolds, a pyridine group was replaced by a benzyl group or a fluorobenzyl group. In the pyridine variant, the pyridine nitrogen forms a water-mediated bridge to a glycine residue deep within the binding pocket. This water-mediated contact seems to be critical for the stability of the inhibitor in the binding pocket and is present in most of the active compounds in this study. When a fluorobenzyl group instead of pyridine is present, the water-mediated contact can still be formed, with water forming a (possible) hydrogen bond to fluorine, but the contact is expected to be much weaker. This would explain the reduced inhibitor activity of the fluorobenzyl variant compared to that of the pyridine variant. By contrast the benzyl variant cannot interact with the water molecule, and the absence of the water-mediated contact likely explains that all benzyl variants of the inhibitor are inactive (half maximal inhibitory concentration >50 μM).

The modulating effect of fluorine on a complex hydrogen bonded water network inside a protein environment can also be observed in the case of the μ opioid receptor. The μ opioid receptor is a membrane-bound G protein-coupled receptor, which exhibits a water network that stretches across the receptor. Lešnik et al.^24^ studied the response of this water network to a fluorinated ligand. Specifically, they compared the unfluorinated ligand fentanyl and the fluorinated ligand NFEPP, which is identical to fentanyl except for a single fluorine substitution. They found that the fluorinated ligand induces a markedly different protein–water hydrogen bond network than the unfluorinated fentanyl. These changes in the water network are relevant for drug design, since agonists of μ opioid receptor that selectively bind at low pH values are highly sought after.

Fluorination can also affect the binding affinity of protein ligands through entropic effects. Breiten et al.^26^ study a series of similar ligands that differ by fluorine substitution in their binding thermodynamics to human carbonic anhydrase. The ligands show a very similar binding affinity, but the enthalpic and entropic contributions to this binding affinity widely vary across the ligands. Using Inhomogeneous Solvation Theory^80,81^ based calculations, these effects can be linked to disruptions in the water network in the binding site, where the fluorine disrupts the water network in a way that causes less restricted water motion. This highlights not only how fluorine can be added to a protein binding ligand to specifically modulate a water network but also how difficult it may be to predict the effect of fluorination to the binding strength of a protein ligand and that considerable efforts, involving theory, are needed to rationalize and quantify the specific effects, as they can vary in unexpected ways.

Ye et al.^23^ demonstrated in inhibition assays that fluorination of the unnatural amino acid α-butyric acid in direct proximity of a water filled binding pocket in a protein–protein complex can restore inhibitor activity. Following the hypothesis that the restoration in inhibitor strength is driven by the fluorine binding to the water network in the binding pocket, eventually establishing a water mediated bond to the protein, we investigated the water network of the complex and its interaction with the fluorinated amino acid using molecular dynamics simulations.^82,83^ While we found the water molecules in the binding pocket to be highly connected and binding to the protein receptor, we did not observe any hydrogen-bond-like interactions with fluorine as acceptor.

Hydrogen bonds with fluorine as an acceptor rarely seem to be the driver of ligand binding affinity. Rather, fluorine substituents modulate complex molecular structures like protein–water hydrogen bond networks and thereby may stabilize or destabilize a ligand–protein complex. To disentangle and rationalize the various effects of fluorine substituents in the ligand–protein system, detailed computational models are essential. This is particularly true if the fluorine substituent influences water networks at the interface between the ligand and protein. Reference (84) reviews recent computational methods to analyze protein–water interactions.

### Entropic Effects

A direct consequence of the observation that the effects of fluorine substituents usually cannot be rationalized in terms of simple enthalpic effects is that entropic and, in particular, enthalpy–entropy compensation play a crucial role. We already touched on this topic in the context of fluorinated human carbonic anhydrase ligands and their effect on the water network but will discuss it in more detail in this section.

A recent example for enthalpy–entropy compensation in a fluorinated system is the ligand binding to STING protein studied by Smola et al.^85^ The authors compared the binding free energies of fluorinated and unfluorinated cyclic dinucleotides with isothermal calorimetry and computational QM and QM/MM calculations. The fluorinated ligands show more favorable binding entropy because of lower conformational flexibility in the unbound state and less entropic cost due to interactions with solvent and receptor. This effect is partially compensated by stronger enthalpic interactions of the unfluorinated ligands.

**Figure 7:**
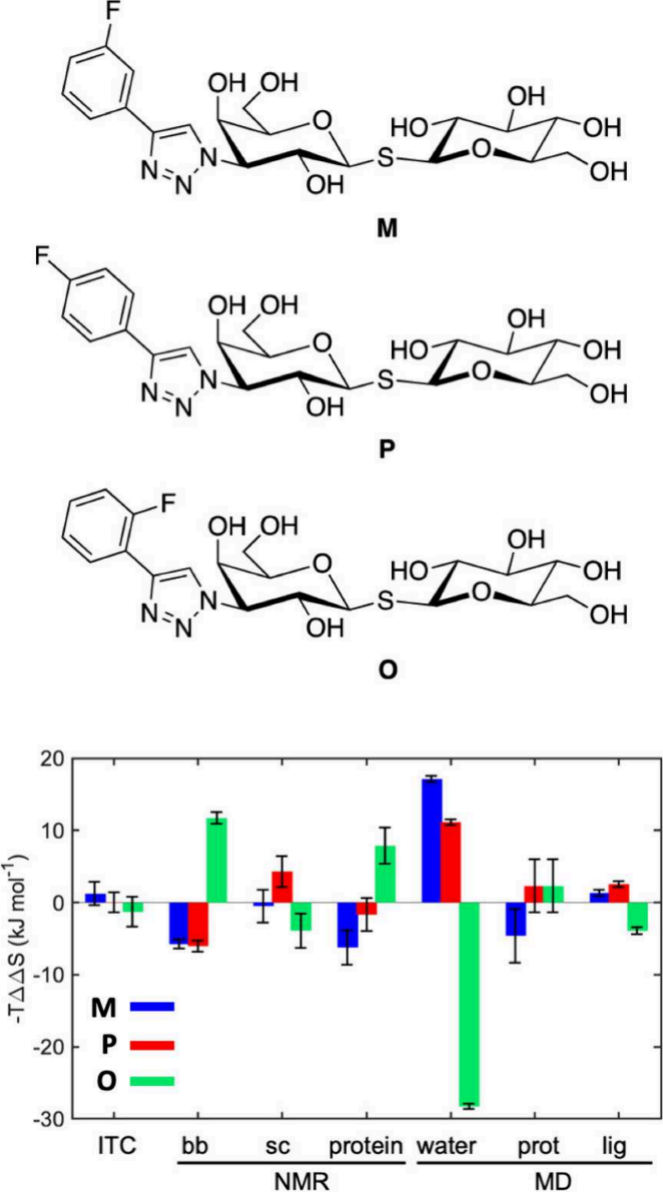
Entropic contributions to binding free energy of ligands **M**, **P** and **O**. −*TΔΔS* is the difference of the entropic contribution to ligand binding, and −*TΔS* is between one complex and the average contribution of the two other complexes. Adapted from ref (25). Available under a CC BY license. Copyright 2021 Wallerstein et al.

Fluorinated systems exhibit not only enthalpy–entropy compensation but also entropy–entropy compensation, in which different kinds of entropy, i.e., protein conformational entropy, ligand conformational entropy, and solvation entropy compensate each other. Wallerstein et al.^25^ found entropy–entropy compensation in the protein–ligand systems of galectin-3 in complex with three different fluorinated ligands, which only differed in the position of fluorine on the phenyl ring of the ligand (*o*-, *m*-, or *p*-fluorophenyl, Figure 7). The authors study the binding thermodynamics of the three systems using multiple experimental and computational methods like X-ray crystallography, isothermal titration calorimetry, NMR relaxation, Molecular Dynamics, and Grid Inhomogeneous Solvation Theory (GIST).^80,81^ They found that the overall entropic contribution to binding is about equally strong in all three ligands. However, when the entropic contribution is decomposed into various contributions, using either NMR or MD simulations, each ligand exhibits a unique pattern of opposing entropic effects. Many of the entropic effects have a sizable magnitude. Specifically, the *o*-substituted ligand stands out from the other two ligands and is almost entirely stabilized in the complex by the change in water entropy. The study showcases that detailed computational and experimental analysis may be needed to understand the influence of such a minor change in fluorination as the repositioning of a single fluorine on the overall binding strength of a fluorinated ligand.

Finally, we want to note that fluorination can also give rise to compensating enthalpic effects and unexpected trends in physicochemical properties like hydration free energy of fluorinated amino acids.^35^ Compensating enthalpic effects may originate in changes in surface area, disruptions of backbone–water hydrogen bonds, and changes in side chain polarity. Delineating and quantifying these effects separately can be achieved by alchemical free-energy perturbation.^86,87^

## Conclusions

Fluorine is a powerful and versatile modulator of molecular interactions through its unique properties. However, the effects of fluorination of such ligands can often be unexpected and difficult to rationalize. The support of computational methods is essential for making rational predictions about the effects of fluorination. In this Perspective, we covered different aspects of how computational methods help understand fluorine in protein binding ligands, including accurate force fields, hydrogen bond interactions, aryl groups, water networks, and entropic effects. By showcasing the variety of fluorine interactions, we hope to provide a resource for researchers who design fluorinated protein–ligand complexes. Our objective of this work is to provide clarity on what aspects to investigate and on computational methods to quantify these aspects.
